# Transgenic cotton and farmers’ health in Pakistan

**DOI:** 10.1371/journal.pone.0222617

**Published:** 2019-10-02

**Authors:** Shahzad Kouser, David J. Spielman, Matin Qaim

**Affiliations:** 1 Department of Economics, COMSATS University Islamabad, Islamabad, Pakistan; 2 Environment and Production Technology Division, International Food Policy Research Institute, Washington, DC, United States of America; 3 Department of Agricultural Economics and Rural Development, University of Goettingen, Germany; Kansas State University, UNITED STATES

## Abstract

Despite substantial research on the economic effects of transgenic insect-resistant *Bacillus thuringiensis* (Bt) cotton, there is still limited work on this technology’s impacts on human health. Due to the inbuilt insect resistance, Bt cotton requires fewer pesticide sprays than conventional cotton, which is not only advantageous from economic and environmental perspectives, but may also result in health benefits for farmers. Using socioeconomic and biophysical data from Pakistan, we provide the first evidence of a direct association between Bt gene expression in the plant and health benefits. A key feature of this study is that Bt cotton cultivation in Pakistan occurs in a poorly regulated market: farmers are often mistaken in their beliefs about whether they have planted Bt cotton or conventional cotton, which may affect their pesticide-use strategies and thus their pesticide exposure. We employ a cost-of-illness approach and variations in the measurement of Bt adoption to estimate the relationship between Bt cotton and farmers’ health. Bt adoption based on farmers’ beliefs does not reduce the pesticide-induced cost of illness. However, adoption based on measuring Bt gene expression is associated with significant health cost savings. Extrapolating the estimates for true Bt seeds to Pakistan’s entire Bt cotton area results in annual health cost savings of around US$ 7 million. These findings have important implications for the regulation of seed markets in Pakistan and beyond: improved regulations that ensure claimed crop traits are really expressed can increase the benefits for farmers and society at large.

## Introduction

During the past 20 years, the widespread adoption of transgenic cotton by farmers in many different countries has attracted considerable attention. Transgenic cotton with genes from *Bacillus thuringiensis* (Bt) that produce δ–endotoxins protect the plant against *Lepidopteran* insect pests. Bt cotton is commonly cultivated to protect against bollworms that infest close to 90% of the global area under cotton and cause significant crop losses [1]. Bt cotton adoption has generated sizeable economic gains by reducing pesticide costs and increasing effectively-harvested yields [2–6].

In addition to these economic gains, Bt cotton may generate environmental and health benefits by reducing the need for chemical pesticide sprays [7]. Chemical pesticides are known to be associated with negative externalities, including damage to beneficial insects and biodiversity [8–11], pollution of soil and water resources [12], and health problems for farmers and consumers [13–15]. Negative health effects of pesticides for farmers can be particularly severe in developing countries, where pesticide regulations are often enforced with less vigor than in industrialized countries and where pesticides are typically sprayed manually with limited protective clothing [16–19]. Acute, short-term symptoms of exposure to chemical pesticides include respiratory, gastrointestinal, allergic, and neurological disorders, among others.

Several studies have documented a lower incidence of acute pesticide poisoning symptoms among cotton farmers that adopted Bt technology. For instance, research in China [20–21] and South Africa [22] showed that Bt cotton adopters suffered from fewer pesticide poisoning incidences than non-adopters of Bt technology. However, these early studies simply compared mean poisoning incidences reported by Bt and non-Bt farmers without adequately controlling for possible confounding factors. Subsequent studies used more sophisticated statistical approaches. Hossain et al. [23] used regression models to show that Bt adoption reduced the incidence of pesticide poisoning in China, even after controlling for other observable factors. Kouser and Qaim [24] used panel data and statistical differencing techniques to confirm the health benefits of Bt cotton adoption in India. But a common drawback of these studies is that they simply count the poisoning incidences without considering the severity of the symptoms. Some of the symptoms may be minor, so that farmers would not pursue any medical treatment [25–26]. In other cases, the symptoms may be more severe, such that farmers decide to consult a doctor, take medications, or at least rest for a while to recover. Hence, simply counting the incidences may not necessarily lead to good estimates of the actual health costs incurred.

Here, we add to this body of research by using a cost-of-illness approach to evaluate the health benefits of Bt adoption. The cost-of-illness approach considers both the direct and indirect costs of acute symptoms associated with pesticide exposure [18]. We use a double-hurdle model to explain in a first step whether cotton farmers experienced any pesticide-related symptoms during or after spraying. In a second step, the cost-of-illness is then estimated in monetary terms.

Another contribution to this body of research is the careful look we take at the Bt adoption variable itself. Previous studies analyzing the health effects defined Bt adoption as a simple dummy variable based on farmers’ self-reported adoption status (their beliefs about using Bt seeds). Farmers’ beliefs may be a good reflection of actual technology adoption in well-functioning and regulated seed markets, but not necessarily in less formal and poorly regulated seed markets. At the time of seed purchase, Bt has credence attributes. Hence, in poorly regulated markets, there may be cases where farmers believe they have purchased Bt seeds when in fact they have not. Uncertainty about the quality of Bt seeds was reported for several developing countries, including China, India, and Pakistan [4, 27–31]. Low-quality Bt seeds may lead to lower pesticide reduction than what is possible with higher-quality seeds, implying that the health benefits would be underestimated when simply relying on farmers’ self-reported adoption status. We avoid this problem by using biophysical survey data on actual Bt gene expression in plant tissue samples taken from farmers’ fields and analyzed in the laboratory. Results obtained with this “true” Bt adoption variable are compared with those obtained when using farmers’ self-reported adoption status.

The empirical analysis builds on representative data from cotton farmers in Pakistan. The biophysical data on actual Bt gene expression are combined with data from a comprehensive socioeconomic survey. While the concrete results are specific to Pakistan, the broader findings are also relevant for other developing countries where pesticide and seed market regulations are relatively weak. Several recent studies have pointed out that quality issues in agricultural input markets are commonplace in many developing countries due to information asymmetries between input dealers and farmers [31–33].

Our study also brings nuance to the heated discourse around the introduction of transgenic crops for which a key expected advantage—reduction in pesticide exposure and associated illnesses—has often been viewed as a second-order benefit behind better control of crop damage and reductions in the financial cost of spraying. The moratorium on the introduction of Bt eggplant in India in 2010, based largely on non-scientific arguments against the technology, underscores the importance of estimating health effects as a first-order concern [34]. The subsequent diffusion of Bt eggplant introduced in Bangladesh in 2014—or other crops embodying similar insect-resistance and pesticide-reducing traits—may hinge precisely on such kind of evidence.

The remainder of this article is structured as follows. In the next section, we provide some background to cotton production and pesticide use in Pakistan. Then we describe the design of the surveys and the empirical models used to explore the impact of Bt cotton on human health. This is followed by presentation of the estimation results and a discussion of policy and research implications.

## Cotton production and pesticide use in Pakistan

Cotton is Pakistan’s most important cash crop, contributing 10% to national gross domestic product and 55% to foreign exchange earnings [35]. Cotton in Pakistan is mainly produced in two provinces, namely Punjab and Sindh, accounting for 80% and 19% of total national production, respectively [35]. Chemical pesticide use was introduced to cotton production in Pakistan following the Green Revolution of the 1970s, and was actively promoted through the public extension service, farmer subsidy schemes, and other interventions. Not surprisingly, farmers quickly found themselves on a pesticide “treadmill” that forced them to combat emerging resistance with a succession of increasingly toxic pesticides such as chlorinated hydrocarbons, organophosphates, and pyrethroids. As a result, the quantities and frequencies of pesticide application have increased over time: between 1991 and 2000, the share of all insecticides used in Pakistan that was applied to cotton increased from 65% to 80% [36–37]. Yet, despite high levels of pesticide use, cotton crop losses—especially those attributable to cotton bollworms—remained high [38–40].

Occupational exposure to hazardous pesticides poses severe health risks to farmers, field workers, and cotton pickers. Existing studies showed that limited knowledge of pesticide safety and poor application practices—such as manual preparation of spray solutions, incorrect dosages, pesticide spills from backpack sprayers, poor spraying technology, and limited protective clothing use—are the main causes of high human exposure in the cotton belts of Punjab and Sindh [40–45]. Several studies have observed significantly increased concentrations of pesticide residues in blood samples of pesticide operators [45–46]. Cotton pickers, often poor female laborers, were also found to suffer from pesticide-related illnesses [47–48]. To reduce health and environmental problems of pesticide use, in the mid-1990s farmer field schools were established in Pakistan’s major cotton-producing districts to promote low-pesticide use and integrated pest management (IPM) techniques [49]. However, due to low literacy rates among farmers and other constraints, IPM adoption has remained low.

Large problems with chemical pesticide use and pest-related crop damage paved the way for Bt technology, which entered Pakistan’s cotton seed market in the mid-2000s, without the requisite biosafety approvals [50–51]. Local varieties containing the MON531 transgenic event (commercially known as Bollgard I, developed by Monsanto) proliferated widely as a means of controlling bollworm infestations [52] and generated significant production benefits [6, 16, 27, 53–54]. Official approval for a set of Bt cotton varieties that were already under cultivation came from the National Biosafety Committee in 2010, with a second batch of approvals following in 2014. Since then, Pakistan’s seed industry has continued to rely on the MON531 event. Newer and more effective events (such as Bollgard II) are not yet commercially available in Pakistan [55].

## Materials and methods

### Socioeconomic and biophysical surveys

Data used in this study originate from two sources. The first is a survey of farm households conducted in 2013–14 by the International Food Policy Research Institute (IFPRI), Washington DC and Innovative Development Strategies (IDS), Pakistan. The second is a biophysical survey conducted during the same period and on the same farms by the University of Agriculture, Faisalabad (UAF) and the National Institute of Genomics and Biotechnology (NIGAB), in conjunction with IFPRI and IDS. The study protocol was reviewed and received approval from the Institutional Review Board (IRB) of IFPRI (IRB No. 00007490; FWA No. 00005121). The complete dataset used in this study is publicly available at https://dataverse.harvard.edu/dataset.xhtml?persistentId=doi:10.7910/DVN/14LFQF. Due to high rates of illiteracy in the study area, verbal informed consent was asked and recorded for all farmers.

Both surveys were conducted in all six cotton-producing agro-ecological zones of Punjab and Sindh and followed a common multistage sampling procedure to select agro-ecological zones, mouzas (villages), and households. In the first stage, six cotton-producing agro-ecological zones were identified in both provinces. In the second stage, 52 mouzas were randomly selected within six agro-ecological zones with probabilities proportional to population sizes. In the last stage, 14 cotton growers were randomly selected with equal probabilities in one randomly selected block within each mouza, yielding a sample of 728 households. The selected households are representative of cotton farmers in these zones.

The household survey was conducted via face-to-face interviews with farmers using a structured questionnaire. These interviews were conducted in three rounds beginning in April 2013, as farmers were preparing their land for cotton cultivation; and culminating in February 2014, immediately after harvest. Data were collected on household social and economic variables such as age, education, and household size; farm variables like farm size, cultivated area, varietal choice, and beliefs about Bt cotton; and input use (seed, fertilizer, agrochemicals, and labor) in cotton production. The input-use modules included questions on the type, formulation, and price of pesticides used; the number and quantity of pesticide applications; the use of protective measures; and who sprayed the crop (farmer or hired laborer). Farmers were also interviewed about the frequency of various pesticide-related acute symptoms such as nausea, dizziness, coughing, muscular twitching, tremor, vomiting, abdominal cramps, diarrhea, blurred vision, and eye and skin irritations experienced during the 2013 cotton growing season. For farmers who suffered from these symptoms, additional questions were asked about direct expenses such as self-treatment costs, consultation costs of physicians, medication costs, travel costs to and from health facilities, and indirect costs such as work days lost. While farmers may also use small quantities of pesticides on crops other than cotton, cotton is clearly the main cash crop for all farmers in our sample, accounting for the lion’s share of all pesticide applications. Hence, it is reasonable to assume that pesticide-related health symptoms are primarily due to cotton spraying.

The biophysical survey was conducted in two rounds: at 70 days after sowing (DAS) (June-August 2013) and at 120 days after sowing (August-October 2013). During each round, cotton leaves and bolls were collected from five randomly chosen plants located in the sample household’s main plot. The tissue samples were tested for (1) the presence of Bt genes, using lateral flow strip assays (QuickStix Combo Kits, which we refer to as strip tests) manufactured by EnviroLogix Inc., and (2) the expression levels of Bt protein, using ELISA kits (QualiPlate Combo Kit for Cry1Ac and Cry2Ab) from the same manufacturer.

By the third round of the household survey and the second round of the biophysical survey, sample attrition was non-trivial, although there is no evidence of attrition bias. After matching the biophysical data with the socioeconomic data, the final sample used in our study contained data for 564 cotton farmers.

### Cost-of-illness approach

Earlier studies that investigated the effects of chemical pesticide applications on farmers’ health have used the number of reported poisoning incidences as a proxy for ill health [23–24, 56]. However, simply adding up all reported incidences may be misleading, as some of the incidences may only be associated with minor symptoms that do not require medical treatment. Other symptoms may be associated with significant health costs. The cost-of-illness approach is better able to capture such differences, as it assigns a zero value to symptoms for which farmers did not require any treatment. In this approach, treatment is not confined to actual medication or consulting a doctor, but also includes lost work time due to ill health.

The cost-of-illness approach has been extensively employed in the study of health costs incurred by pesticide exposure, pollution, food poisoning, and water contamination [57–59]. This approach allows for the estimation of the direct and indirect costs of pesticide-related acute symptoms [59]. Direct costs are estimated as the sum of a farmer’s self-reported costs of doctor consultation, travel, medication, and home-based health care for all above-mentioned acute symptoms. Indirect costs include the opportunity cost of work days lost by farmers due to illness. The opportunity cost of work days lost was valued at the current wage rate locally paid to farm workers at the survey time, taking into account individual skills. It is worth noting that health costs based on this cost-of-illness approach provide a lower bound estimate of the true costs of ill health, as other potential costs, such as the opportunity cost of work days lost by nursing household members, work productivity losses, the value of foregone leisure, or defensive expenditures, are ignored [25]. Furthermore, the health costs occurring to farm workers are ignored, as we only interviewed farmers. Contingent valuation methods are an alternative technique that is useful to also value intangible costs, such as pain, discomfort, stress and suffering [60–62]. However, contingent valuation methods are associated with hypothetical bias, while the cost-of-illness approach has the advantage that it is based upon real market conditions [18].

### Modeling the association between Bt cotton adoption and farmers’ health

This study models a farmer’s cost of illness from pesticide exposure as a two-stage process. The first stage explains whether or not a farmer experienced one or more poisoning incidences during the last growing season with a severity that required medical treatment. The second stage explains the treatment costs in monetary terms conditional on the first-stage outcome being positive. Following Wooldridge [63], the first-stage binary choice model can be expressed as:

$$dh_{i}^{*}=\alpha x_{i}+\mu_{i}:\;\mu_{i}∼N\left(0,\;1\right)\;and\;dh_{i}=\{\begin{array}{c} 1\;if\;dh_{i}^{*}>0\\ 0\;otherwise \end{array}$$ (1)

where ${dh}_{i}^{*}$ is a latent variable for *dh*_*i*_, which is equal to one if farmer *i* incurs any medical costs to treat acute pesticide-related symptoms, and zero otherwise. The vector of covariates is denoted by *x*_*i*_. Similarly, the second stage decision can be described as:

$$Qh_{i}^{*}=\beta z_{i}+\nu_{i}:\;\nu_{i}∼N\left(0,\;\sigma^{2}\right)\;and\;Qh_{i}=\{\begin{array}{c} Qh_{i}^{*}\;if\;Qh_{i}^{*}>0\;and\;dh_{i}=1\\ 0\;otherwise \end{array}$$ (2)

where ${Qh}_{i}^{*}$ is a latent variable for *Qh*_*i*_ indicating the monetary cost of treatment (cost of illnesses) for farmer *i*. *z*_*i*_ denotes the vector of covariates, which can overlap with *x*_*i*_. The vectors of parameters to be estimated are denoted as *α* and *β*, while *μ*_*i*_ and *ν*_*i*_ are random error terms. We are particularly interested in the parameters associated with Bt adoption, which is included in both vectors *x*_*i*_ and *z*_*i*_. We hypothesize that Bt adoption reduces chemical pesticide use and is, therefore, negatively associated with the probability of requiring medical treatment and the cost of illness incurred by farmers.

To account for the fact that self-reported Bt adoption may differ from true adoption, we define Bt adoption in three different ways. First, we use a Bt adoption dummy variable based on farmers’ self-reported adoption status. Second, we use Bt adoption dummies based on the laboratory tests of the presence or absence of the Bt gene in the plant tissue samples. Third, we use a continuous Bt variable based on laboratory tests of the Bt toxin expression level. Eqs (1) and (2) are estimated three times, separately for each of the Bt adoption definitions.

The choice of other covariates included in *x*_*i*_ and *z*_*i*_ is based on the extant literature [18, 24, 64]. Not all farmers in the sample sprayed chemical pesticides themselves: in some cases the spraying was delegated to farm workers. Therefore, we use a self-spray dummy variable to account for systematic differences in pesticide exposure. Nevertheless, even when a farmer does not spray himself/herself, he/she may still be exposed to pesticides during monitoring [41], which is why we include the observations from all cotton farmers in the estimates. In addition to the self-spray dummy variable, we control for the number of protective measures used (long-sleeved shirts, gloves, masks, and closed shoes) during spraying. Furthermore, socioeconomic characteristics, such as farmers’ age, education, and off-farm employment, are included. Age and education are assumed to be negatively correlated with health cost [65]. We also include a dummy variable “SC habits” to capture habits related to smoking and chewing tobacco or *paan* (betel leaves). Smoking and chewing during spraying operations increase the risk of inhaling or ingesting toxic vapor. Finally, variables that control for climatic and other regional variations are also included.

### Double-hurdle model

The cost-of-illness variable has a zero-inflated characteristic as some farmers do not pursue any medical treatment. Hence, Eq (2) can result in corner solutions in a utility-maximizing model. The Tobit model is often used to account for censoring of the dependent variable [63]. However, the Tobit model has limitations because it considers constant relative partial effects for a pair of explanatory variables. Furthermore, it assumes that the outcomes—whether and how much to spend for medical treatment—are determined by the same factors. To overcome these restrictive assumptions, we use Cragg’s double-hurdle (DH) model to estimate Eqs (1) and (2) [66]. The DH model is a flexible two-stage model that allows the outcomes in both stages to be determined by different covariates. Its application to problems similar to ours is common in the agricultural economics literature [67–74].

We use the DH model and a likelihood specification as described by Jones [75], which follows the functional forms given in Eqs (1) and (2):

$$L\left(Qh_{i}|x_{i},0\right)=\left\{\prod_{Qh_{i}=0}\left[1-\Phi \left(\gamma \;x_{i}/\sigma_{\mu }\right)\right]\Phi \left(\beta z_{i}/\sigma_{v}\right)\right\}\times \left\{\prod_{Qh_{i}>0}\Phi \left(\gamma \;x_{i}/\sigma_{\mu }\right)\Phi \left(\beta z_{i}/\sigma_{v}\right)\right\}\times \left\{\frac{\phi \left[Qh_{i}-\beta z_{i}\right]/\sigma_{v}}{\sigma_{v}\Phi \left(\beta z_{i}/\sigma_{v}\right)}\right\}$$ (3)

where *ϕ* and Φ denote the standard normal probability and cumulative distribution functions, respectively. Similarly, *σ*_*μ*_ and *σ*_*v*_ are the standard deviations of *μ*_*i*_ and *ν*_*i*_, respectively. Eq (3) can be solved for *γ*, *β*, and *σ*^2^ through maximum likelihood estimation.

The Tobit estimation is nested in the DH model. A likelihood ratio (LR) test is used to test which of the two specifications is more appropriate. The log-likelihood of the DH model consists of the summation of the log-likelihood values estimated in the first hurdle by a probit regression, and in the second hurdle by truncated regression estimators. We discuss the test results below.

### Calculating marginal effects

The coefficient estimates from the DH model are useful to interpret the sign and levels of significance, but marginal effects are more suitable to interpret the magnitude of the effects. Conditional average marginal effects (CAME) of each covariate can be calculated following Burke [76]. Based on the first-hurdle estimates, we calculate the probability of farmer *i* requiring medical treatment as:

$$P({dh}_{i}^{*}>0|x_{i})=\Phi (\gamma x_{i})$$ (4)

$$P({dh}_{i}^{*}=0|x_{i})=1-\Phi (\gamma x_{i})$$ (5)

Then, given *Qh*>0, the conditional health cost for each farmer *i* is estimated as:

$$E({Qh}_{i}{|Qh}_{i}>0,z_{i})={\beta z}_{i}+\sigma \times \lambda (\beta \;z_{i}/\sigma )$$ (6)

where *λ*(*βz*_*i*_/*σ*) = *ϕ*(*β z*_*i*_/*σ*)/Φ(*β z*_*i*_/*σ*) is the inverse Mills ratio.

The unconditional average marginal effects (UAME) can be calculated by uniting the effects of both hurdles as:

$$E({Qh}_{i}|x_{i},z_{i})=\Phi (\gamma x_{i})[\beta z_{i}+\sigma \times \lambda (\beta z_{i}/\sigma )]$$ (7)

Interpretation of UAME is particularly useful for policy-making purposes.

## Results and discussion

### Self-reported adoption and biophysical analysis

Table 1 compares farmers’ self-reported Bt adoption with the strip test results for the presence of the Bt gene and the ELISA test results for Bt gene expression levels. In Punjab, 81% of the sample farmers classified themselves as Bt adopters, whereas for 82% of the farmers the strip test showed the presence of the Bt gene in at least one of the tissue samples taken. At first glance, this suggests close correspondence in adoption rates. In reality, however, sizeable errors occur in various directions. Approximately 17% of the farmers in Punjab that reported having used Bt seeds had actually not used true Bt seeds: this is referred to as type I error [29, 77]. Conversely, for 57% of the farmers that reported having used non-Bt seeds, the strip tests indicated positive results: this is referred to as a type II error. Type I error may be due to deliberately deceptive marketing practices by seed producers or retailers. The occurrence of type II errors, on the other hand, suggests that there are also more general seed quality uncertainties in these poorly regulated seed markets. This is supported by the fact that about 12% of the farmers in Punjab were unsure whether or not they had planted Bt seeds. Overall, 30% of the farmers in Punjab were either incorrect in their beliefs (Type I or Type II errors) or uncertain and these farmers have applied the largest quantities of pesticides in the whole sample.

**Table 1:** Self-reported Bt adoption and results from laboratory analysis on Bt expression by province.

Self-reported adoption status	Variables	Punjab province			Sindh province		
		Bt presence (based on strip test) ^a^			Bt presence (based on strip test)		
		Total	All negative	At least one positive	Total	All negative	At least one positive
Bt	Observations	353	61	292	50	1	49
	Bt toxin (μg/g)	0.96	0.48	1.18	2.51	0	2.57
	Pesticide quantity (kg/acre)	2.47	2.92	2.04	2.12	1.13	2.14
Non-Bt	Observations	30	13	17	28	14	14
	Bt toxin (μg/g)	0.74	0.56	0.88	1.25	0.23	2.26
	Pesticide quantity (kg/acre)	2.64	2.78	2.53	1.94	2.32	1.55
Don’t know	Observations	51	6	45	51	17	34
	Bt toxin (μg/g)	1.14	0.25	1.26	1.49	2.20	2.23
	Pesticide quantity (kg/acre)	1.88	3.25	1.69	2.01	0	1.92
No response	Observations	1	-	1	-	-	-
	Bt toxin (μg/g)	0.68	-	0.68	-	-	-
	Pesticide quantity (kg/acre)	2.3	-	2.3	-	-	-
Total	Observations	435	80	355	129	32	97
	Bt toxin (μg/g)	0.97	0.48	1.08	1.84	0.10	2.41
	Pesticide quantity (kg/acre)	2.41	2.93	2.30	2.04	2.22	1.98

^a^ Due to logistical constraints, only two of the five plant tissue samples taken from farmers’ fields could be lab-tested in Punjab province.

In Sindh, only 39% of the farmers believed that they had planted Bt seeds, whereas 75% actually had based on the strip test results (type II error). The occurrence of type I errors was much lower in Sindh than in Punjab. Overall, 51% of the farmers in Sindh were either incorrect in their adoption beliefs or uncertain. On average, intentional Bt adoption and also pesticide use levels are somewhat lower in Sindh than in Punjab, which is due to regional differences in bollworm infestation rates.

In addition to the strip tests, we used ELISA tests to determine the actual level of Bt toxin expression in the plant. These results are also shown in Table 1, based on the samples taken 70 days after sowing. It should be noted that the strip tests may conclude the absence of the Bt gene when expression levels are low, which is why mean expression levels are positive in some of the cases even when the strip tests were negative. In general, the higher the Bt expression the more effective is the bollworm control. Hence, we expect that Bt toxin expression is negatively associated with pesticide use and related health costs.

### Descriptive statistics of key variables

Table 2 presents descriptive statistics of other variables used in the regression analysis by self-reported Bt adoption status. Farmers who were uncertain about the nature of their seeds are classified as non-adopters in this classification. Bt adopters and non-adopters do not differ significantly in terms of age, education, and most other socioeconomic variables.

**Table 2:** Descriptive statistics by self-reported Bt adoption status.

Variables	Unit	Bt adopters (N = 403)	Non-Bt adopters (N = 161)
Household characteristics			
Age	Year	46.53 (11.59)	45.95 (12.09)
Education	Year	4.90 (4.39)	4.27 (4.36)
Household size	Members	8.94 (4.62)	8.94 (4.34)
Off-farm employment	Dummy	0.20 (0.40)	0.19 (0.40)
Farm and farm management variables			
Farm size	Acre	9.08 (17.48)	6.64 (13.19)
Cotton area	Acre	5.99 (13.46)	4.30 (8.89)
Bt toxin expression	μg/g	1.16 (1.05)	1.19 (1.32)
Pesticide exposure and health related variables			
Pesticide quantity	Kg/acre	2.43^*^ (2.28)	2.08 (1.33)
Pesticide cost	Rs/acre	4173.86 (6433.56)	3345.70 (2850.17)
Self-spray	Dummy	0.58^*^ (0.50)	0.65 (0.48)
Total protective gears worn	No.	1.86 (1.78)	1.74 (1.63)
SC habits	Dummy	0.45^***^ (0.50)	0.31 (0.46)
Medical treatment	Dummy	0.78 (0.42)	0.77 (0.42)
Cost of illness	Rs./season	289.45 (255.84)	293.82 (224.95)

Notes: Mean values are shown with standard deviations in parentheses.

^***^ and ^*^ indicate that the mean values between self-reported Bt adopters and non-Bt adopters are significantly different at the 1%, and 10% levels, respectively.

t-tests are used for continuous and chi-square tests are used for categorical variables to identify differences in mean values.

Pesticide-related variables are shown in the lower part of Table 2. Mean pesticide quantity is higher for Bt adopters than for non-adopters. This is surprising and contradicts earlier studies on the effects of Bt cotton. However, as discussed above, due to poorly regulated seed markets in Pakistan, reported Bt adoption does not always mean true adoption of Bt seeds with high levels of toxin expression.

**Fig 1:**
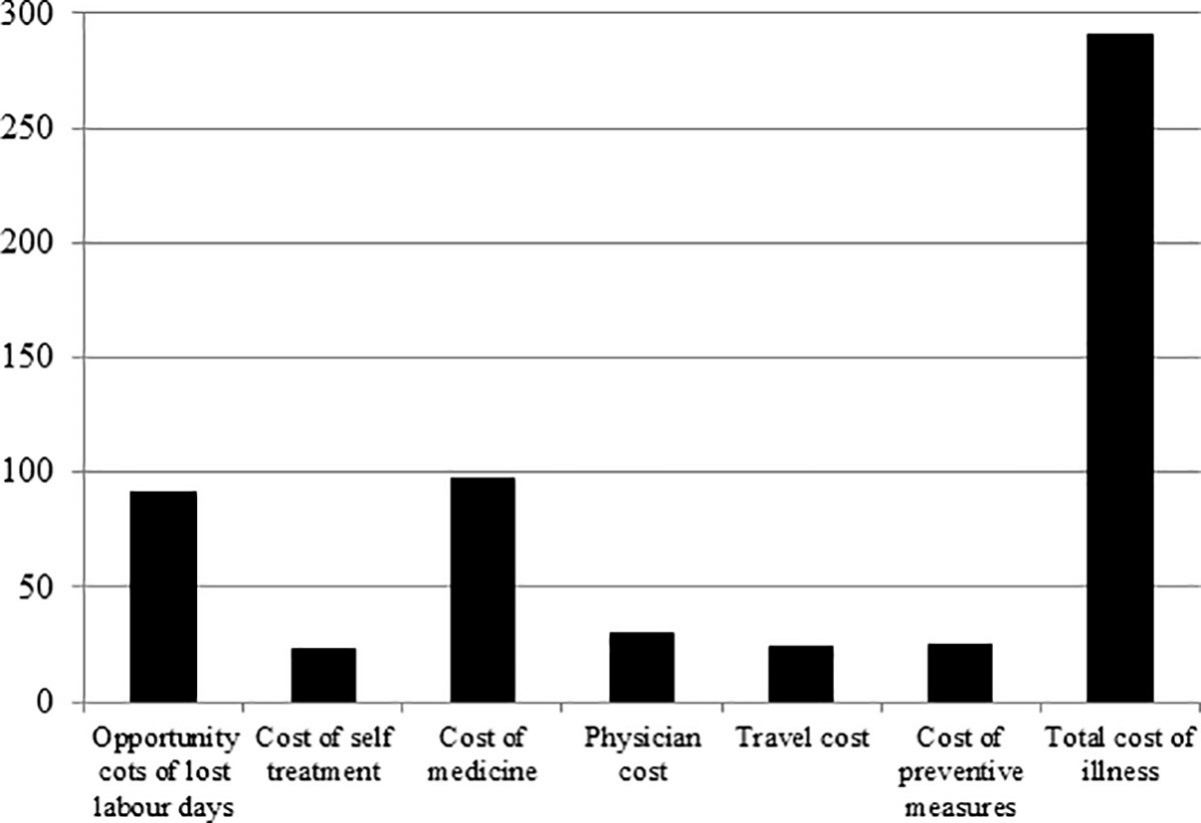
Composition of average cost of illness reported by farmers (in Rs).

**Fig 2:**
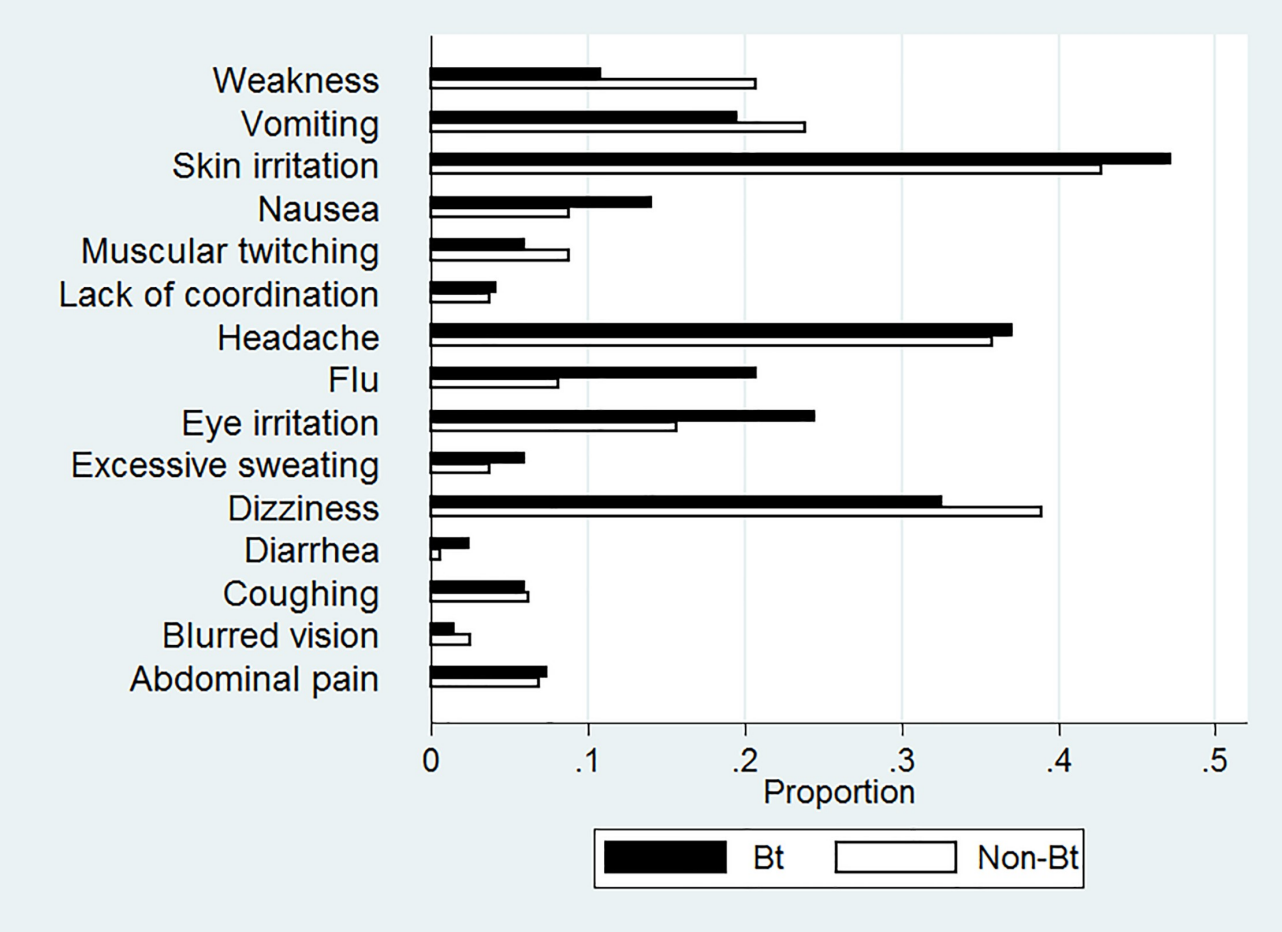
Pesticide-induced acute symptoms experienced by farmers (by self-reported Bt adoption status).

Neither the likelihood of experiencing pesticide-related symptoms that require medical treatment nor the medical treatment costs differ significantly between self-reported Bt adopters and non-adopters. Mean treatment cost incurred by sample farmers during the last cotton growing season was in a magnitude of Rs 290 per cotton season (1 Pakistani rupee (Rs) was 0.00995 US$ in 2014), which is in line with previous studies [7, 42]. The composition of the average cost of illness is given in Fig 1. Further details of the types of acute health symptoms experienced by farmers are shown in Fig 2. Skin irritation, dizziness and headache are commonly reported symptoms by farmers.

Table 3 also shows descriptive statistics but this time differentiating between Bt adopters and non-adopters based on the laboratory tests. We define three categories of farmers: non-Bt adopters are those for whom the strip test results for the samples taken 70 days after sowing were all negative; weak-Bt adopters (or adopters of weakly performing Bt technology) are those with at least one strip test having been positive but Bt expression levels lower than 1.90 μg/g; true-Bt adopters are those with at least one strip test having been positive and Bt expression levels greater than 1.90 μg/g. The threshold of 1.90 μg/g was suggested by various studies as the level required for effective bollworm control [29, 78–80].

**Table 3:** Descriptive statistics by laboratory-based definition of Bt adoption.

Variables	Unit	(1) Non-Bt adopters (N = 112)	(2) Weak-Bt adopters (N = 356)	(3) True-Bt adopters (N = 96)
Household characteristics				
Age	Year	46.09 (1026)	46.95 (12.20)	44.53 (11.46)
Education	Year	3.52 (4.23)	4.90 (4.31)^***^	5.48 (4.63)^***^
Household size	Members	9.04 (4.22)	9.08 (4.76)	8.34 (4.02)
Off-farm employment	Dummy	0.18 (0.39)	0.22 (0.41)	0.15 (0.36)
Farm and farm management variables				
Farm size	Acre	8.04 (13.66)	8.65 (14.32)	7.82 (24.67)
Cotton area	Acre	5.37 (7.71)	5.67 (12.70)	5.10 (15.20)
Bt toxin expression	μg/g	0.37 (0.47)	0.90 (0.47)^***^	3.09 (1.31)^***^
Pesticide exposure and health related variables				
Pesticide quantity	Kg/acre	2.73 (1.84)	2.29 (2.12)^**^	1.98 (2.05) ^***^
Pesticide cost	Rs/acre	4521.57 (5750.13)	3917.24 (5679.18)	3330.95 (5452.26)^*^
Self-spray	Dummy	0.58 (0.50)	0.58 (0.50)	0.69 (0.47)
Total protective gear worn	No.	1.55 (1.57)	1.87 (1.80)^*^	1.97 (1.71)^*^
SC habits	Dummy	0.30 (0.46)	0.44 (0.50)^**^	0.45 (0.50)^**^
Medical treatment	Dummy	0.77 (0.42)	0.68 (0.47)^*^	0.63 (0.49)^**^
Cost of illness	Rs/season	381.10 (274.28)	287.98 (237.87)^***^	195.31 (193.84)^***^

Notes: Mean values are shown with standard deviations in parentheses. Asterisks in columns (2) and (3) show significant differences of variables between weak-Bt adopters and non-Bt adopters and between true-Bt adopters and non-Bt adopters, respectively, with ^***^, ^**^, and ^*^ denoting significance at the 1%, 5%, and 10% level, respectively.

As one would expect, non-Bt adopters applied significantly higher quantities of pesticide than weak-Bt or true-Bt adopters. The results in Tables 2 and 3 suggest that farmers do not decide on pesticide applications simply based on their Bt adoption beliefs but based on actual pest infestation levels observed in the field. Naturally, pest infestation observed in the field is higher with low Bt expression in the cotton plants.

### Double-hurdle estimates

Table 4 reports results from the LR test used to assess the appropriateness of the DH model against the more restrictive Tobit specification. Based on the chi-square test statistics, for all three versions of the Bt adoption variable we reject the null hypothesis that the Tobit specification is appropriate. Hence, we proceed with the DH specification.

**Table 4:** Model specification tests.

	Model (I)	Model (II)	Model (III)
	Self-reported Bt adoption	Adoption based on lab tests	Bt expression levels
Log-likelihood of Tobit regression	-2934.35	-2927.68	-417.77
Log-likelihood of probit regression	-261.30	-256.31	-36.70
Log-likelihood of truncated regression	-2553.78	-2547.38	-355.22
χ^2^ (9/10/9)	257.47	247.97	51.69
*p*-value	0.00	0.00	0.00

Table 5 presents the DH estimation results for all three models with the different Bt adoption definitions. Model (I) uses the farmers’ self-reported Bt adoption status as an explanatory variable, but this does not have a significant effect on either the likelihood of medical treatment (hurdle 1) or the monetary cost of illness (hurdle 2). These estimates contradict findings from previous studies on health effects of Bt cotton adoption [23–24, 64]. However, as shown above, self-reported adoption does not always correspond to true adoption of effective Bt seeds.

**Table 5:** Factors influencing farmers’ health costs (double-hurdle models).

Variables	Model (I) Self-reported Bt adoption		Model (II) Adoption based on lab tests		Model (III) Bt expression levels	
	Hurdle 1	Hurdle 2	Hurdle 1	Hurdle 2	Hurdle 1	Hurdle 2
Bt adoption (dummy) ^a^	-0.15 (0.16)	6.69 (18.91)	-	-	-	-
Weak-Bt adoption (dummy) ^b^	-	-	-0.32^*^ (0.18)	-36.46^*^ (19.43)	-	-
True-Bt adoption (dummy) ^b^	-	-	-0.68^***^ (0.23)	-95.06^***^ (26.70)	-	-
Bt expression (μg/g)	-	-	-	-	-0.77^***^ (0.20)	-72.06^***^ (24.95)
Cotton area (acres)	-0.00 (0.01)	0.81 (0.63)	0.00 (0.01)	0.76 (0.61)	-0.04 (0.04)	-1.34 (3.03)
Self-spray (dummy)	1.26^***^ (0.21)	-48.61^**^ (23.58)	1.29^***^ (0.21)	-47.54^**^ (23.16)	2.70^***^ (0.75)	-144.07^***^ (51.54)
Number of protective devises	0.02 (0.06)	-41.70^***^ (6.53)	0.03 (0.06)	-40.46^***^ (6.43)	-0.45^**^ (0.19)	-28.97^**^ (13.90)
Off-farm employment (dummy)	0.08 (0.17)	-	0.07 (0.17)	-	0.01 (0.46)	-
SC habits (dummy)	-0.04 (0.13)	-29.25^*^ (16.14)	-0.01 (0.13)	-21.46 (15.82)	-0.16 (0.37)	3.24 (28.52)
Farmer’s age (years)	-0.01^**^ (0.01)	-3.84^***^ (0.71)	-0.01^*^ (0.01)	-3.72^***^ (0.70)	-0.00 (0.02)	-0.77 (1.25)
Farmers’ education (years)	-0.08^***^ (0.02)	-26.80^***^ (2.09)	-0.08^***^ (0.02)	-25.50^***^ (2.08)	-0.10^**^ (0.05)	-21.24^***^ (3.84)
Punjab province (dummy) ^c^	0.46^***^ (0.18)	137.41^***^ (21.84)	0.26 (0.18)	130.49^***^ (20.84)	1.30^**^ (0.57)	64.66^*^ (34.53)
Constant	0.60^*^ (0.33)	711.39^***^ (41.24)	0.82^**^ (0.34)	741.38^***^ (41.30)	2.32^**^ (1.00)	773.55^***^ (99.49)
Sigma	149.83^***^ (5.86)		147.42^***^ (5.75)		99.03^***^ (9.72)	
Wald χ^2^ (9/10/9)	137.57^***^		140.81^***^		25.34^***^	
Observations	564		564		96	

Notes: Coefficient estimates are shown with standard errors in parentheses.

^***^, ^**^, and ^*^ denote significance at the 1%, 5%, and 10% level, respectively.

^a^ The base category is non-Bt based on farmers’ self-reported adoption status.

^b ^The base category is non-Bt seeds.

^c ^The base province is Sindh.

In model (II), we use weak-Bt and true-Bt adoption based on the laboratory tests as two separate dummy variables (with non-Bt as the reference). Both variables have significantly negative coefficients in both hurdles, meaning that weak-Bt and true-Bt adoption is negatively associated with the likelihood of requiring medical treatment and the monetary cost of illness. In model (III), instead of the adoption dummies we use Bt expression levels as a continuous variable, restricting the sample to the true-Bt adopters (those with Bt expression levels above 1.90 μg/g). The results indicate that Bt expression has significantly negative effects in both hurdles.

### Marginal effects

For better interpretation of effect sizes, conditional average marginal effects (CAME) are presented in Table 6. The results for model (II) indicate that weak-Bt adoption reduces the probability of requiring medical treatment by 8 percentage points and the cost of illness by Rs 34. As expected, true-Bt adoption has stronger effects: it reduces the probability of requiring medical treatment by 17 percentage points and the cost of illness by Rs 88. The CAME results for model (III) indicate that an increase by 1 μg/g in Bt expression levels reduces the probability of requiring medical treatment by 16 percentage points and the cost of illness by Rs 61.

**Table 6:** Conditional marginal effects from double-hurdle models.

Variables	Model (II) Adoption based on lab tests		Model (III) Bt expression levels	
	Hurdle 1	Hurdle 2	Hurdle 1	Hurdle 2
Weak-Bt adoption (dummy) ^a^	-0.08^*^ (0.05)	-33.74^*^ (18.97)	-	-
True-Bt adoption (dummy) ^a^	-0.17^***^ (0.06)	-87.96^***^ (22.61)	-	-
Bt expression (μg/g)	-	-	-0.16^*^ (0.10)	-61.07^***^ (23.66)
Cotton area (acres)	0.00 (0.00)	0.71 (0.90)	-0.01 (0.01)	-1.13 (3.32)
Self-spray (dummy)	0.32^***^ (0.05)	-43.99^**^ (20.24)	0.57^*^ (0.33)	-122.10^**^ (53.24)
Number of protective devises	0.01 (0.02)	-37.43^***^ (5.12)	-0.10 (0.08)	-24.55^**^ (10.42)
Off-farm employment (dummy)	0.02 (0.04)	-	0.00 (0.14)	-
SC habits (dummy)	-0.00 (0.04)	-19.86 (13.59)	-0.04 (0.11)	2.75 (28.98)
Farmer’s age (years)	-0.00 (0.00)	-3.44^***^ (0.55)	-0.00 (0.00)	-0.65 (0.87)
Farmers’ education (years)	-0.02^***^ (0.00)	-23.59^***^ (2.22)	-0.02^*^ (0.01)	-18.00^***^ (4.94)
Punjab province (dummy)^b^	0.07 (0.05)	120.74^***^ (21.67)	0.28 (0.18)	54.80^*^ (30.28)

Notes: Marginal effects are shown with bootstrapped standard errors in parentheses.

^***^, ^**^, ^*^ denote significance at the 1%, 5%, and 10% level, respectively.

^a^ The base category is non-Bt seeds.

^b^ The base province is Sindh.

The result that Bt adoption is associated with reductions in pesticide-induced health problems among cotton farmers is in line with earlier research in China [23, 81], India [24], and Pakistan [7]. What the results presented here add to the existing literature is that the health benefits are also reflected in a lower cost of illness, and that the effects only occur with true Bt seeds. In poorly regulated seed markets with uncertainty about the quality of Bt technology, as observed in Pakistan, evaluating impacts based on self-reported adoption data may lead to systematic underestimation.

In addition to CAME, we also computed unconditional average marginal effects (UAME) that incorporate results from both hurdles (Table 7). Weak-Bt adoption is associated with a Rs 42 decline and true-Bt adoption with a Rs 94 decline in the pesticide-induced cost of illness. Relative to the unconditional expected cost of illness of Rs 292, these UAME estimates imply a 14% and 32% reduction in the health costs through weak-Bt and true-Bt adoption, respectively. An increase in Bt expression levels by 1 μg/g decreases the cost of illness by Rs 65.

**Table 7:** Unconditional marginal effects of Bt adoption on farmers’ health costs.

Bt variables	Unconditional expected cost of illness (Rs)	Unconditional average marginal effects
Weak-Bt adoption (dummy) ^a^	292.34	-41.96^**^ (19.15)
True-Bt adoption (dummy) ^a^	292.34	-93.72^***^ (23.05)
Bt expression (μg/g)	199.50	-64.69^***^ (13.46)

Notes: The last column shows marginal effects with bootstrapped standard errors in parentheses.

^***^ denotes significance at the 1% level.

^a ^The base category is non-Bt seeds.

The total Bt cotton area in Pakistan is currently estimated at 7.4 million acres [55]. However, this includes seeds with questionable Bt expression levels. If all the 7.4 million acres were grown with true-Bt seeds, our results suggest that the annual health cost savings could be in a magnitude of Rs 695 million (US$ 6.92 million). Furthermore, true Bt seeds with higher gene expression levels would also lead to more effective pest control and therefore lower crop losses and higher financial savings in pesticide expenditures.

## Conclusions

While research in different countries has shown that Bt cotton adoption reduces farmers’ chemical pesticide use and increases cotton yield and profits, opponents of transgenic technology have raised concerns about potential health and environmental risks. This study contributes to the literature by evaluating the health effects of Bt cotton adoption in Pakistan. Pakistan is an interesting example because widespread adoption of Bt cotton already occurred before this technology was formally approved, resulting in the growth of markets for Bt cotton seed in the absence of regulation and resulting in the spread of Bt seeds with varying levels of Bt gene expression. This characteristic of Pakistan’s cotton seed market has contributed to uncertainty among farmers about the technology’s effectiveness in controlling targeted pests.

To explore these issues, socioeconomic and biophysical surveys were conducted in different agro-ecological zones of Punjab and Sindh provinces. A cost-of-illness approach was used to estimate health costs in monetary terms, and apply this approach in reference to the significant discrepancies found between farmers’ self-reported Bt adoption status and adoption defined based on laboratory analysis of plant tissue samples. Using the self-reported data, Bt adoption has no effect on the cost of illness incurred by farmers. However, the picture changes when using data from laboratory analysis. Even at low and moderate levels of Bt gene expression, technology adoption reduces the cost of illness significantly. The effects are stronger at higher levels of Bt expression. Double-hurdle model estimates suggest that the adoption of true-performing Bt technology reduces the probability of experiencing pesticide-induced health symptoms that require medical treatment by 17% and the cost of illness by Rs 88. Using estimates of unconditional average marginal effects, true-Bt seed adoption decreases farmers’ health costs by 33%. Extrapolating these estimates to the entire Bt cotton area in Pakistan results in annual health cost savings of US$ 6.9 million.

The large health costs associated with chemical pesticide use are in line with previous studies for the cotton sector of Pakistan and elsewhere [7, 18, 20, 42]. Likewise, the finding that Bt cotton adoption can reduce these health costs significantly is in line with earlier research in China and India [23–24]. It should be noted that our results are lower-bound estimates of the health benefits because positive spillovers of Bt cotton to the health of agricultural laborers were not considered. Often, spraying operations are carried out by hired rural workers. Moreover, this study ignores environmental benefits of pesticide reduction [6]. The positive health effects should be taken into account in policy-making for biotechnology and transgenic seeds. However, it is also important to stress that the benefits may not fully materialize in poorly regulated seed markets. Improved regulations that ensure that crop traits and genes are really expressed will increase the technological benefits for sustainable agricultural development [51].

Four limitations of this research should be mentioned. First, the study relies on farmers’ own statements about acute health symptoms and medical treatment costs incurred. Own statements may be subject to measurement error and also include personal elements of how to deal with certain health problems. One farmer may decide to pursue treatment of a certain symptom while another may not. Also, the focus on acute symptoms ignores the fact that pesticide exposure may also contribute to chronic diseases, such as reproductive disorders and cancer. Moreover, the cost of illness approach used in this study does not include intangible costs of pesticide-related illness, such as pain and discomfort [61–62]. Second, the study provides strong evidence of a negative association between Bt adoption and pesticide-induced health costs, but causal interpretations should be made with caution. Technology adoption may be endogenous, which could result in selection bias. It is interesting to note in this respect that farmers in Pakistan face considerable uncertainty about the quality of Bt seeds, such that the adoption of true Bt seeds has a certain component of randomness, which may reduce typical issues of selection bias when evaluating the impact of adoption. Nevertheless, follow-up research with longitudinal data could help to improve the identification strategy. Such research could follow a cohort of cotton-growing households over a longer period of time, collecting blood samples and other medical data and data about Bt technology adoption and pesticide use in regular intervals. Third, the study has focused on the role of Bt technology and has not analyzed the potential of other possible interventions–such as strengthening extension services and supporting the spread of knowledge about pesticide safety–that could also help to reduce pesticide-related health cost [43]. Fourth, this study ignores the health impacts of pesticide reduction on casual workers, especially female workers who are often involved in the cotton harvest [47–48]. Future studies could explore the health effects for the different population groups involved in the cotton sector.
